# Computational Study of Molecular Mechanism for the Involvement of Human Serum Albumin in the Renin–Angiotensin–Aldosterone System

**DOI:** 10.3390/ijms251910260

**Published:** 2024-09-24

**Authors:** Daria A. Belinskaia, Natalia N. Shestakova, Kamila V. Samodurova, Nikolay V. Goncharov

**Affiliations:** Sechenov Institute of Evolutionary Physiology and Biochemistry of the Russian Academy of Sciences, 194223 St. Petersburg, Russia; d_belinskaya@mail.ru (D.A.B.); n_shestakova@list.ru (N.N.S.); kamila_sam22@mail.ru (K.V.S.)

**Keywords:** renin–angiotensin–aldosterone system, angiotensin I-converting enzyme, serum albumin, point mutations, molecular modeling

## Abstract

Human serum albumin (HSA) is an endogenous inhibitor of angiotensin I-converting enzyme (ACE) and, thus, plays a key role in the renin–angiotensin–aldosterone system (RAAS). However, little is known about the mechanism of interaction between these proteins, and the structure of the HSA–ACE complex has not yet been obtained experimentally. The purpose of the presented work is to apply computer modeling methods to study the interaction of HSA with ACE in order to obtain preliminary details about the mechanism of their interaction. Ten possible HSA–ACE complexes were obtained by the procedure of macromolecular docking. Based on the number of steric and polar contacts between the proteins, three leading complexes were selected, the stabilities of which were then tested by molecular dynamics (MD) simulation. Based on the results of MD simulation, the two most probable conformations of the HSA–ACE complex were selected. The analysis of these conformations revealed that the processes of oxidation of the thiol group of Cys34 of HSA and the binding of albumin to ACE can reciprocally affect each other. Known point mutations in the albumin molecules Glu82Lys, Arg114Gly, Glu505Lys, Glu565Lys and Lys573Glu can also affect the interaction with ACE. According to the result of MD simulation, the known ACE mutations, albeit associated with various diseases, do not affect the HSA–ACE interaction. A comparative analysis was performed of the resulting HSA–ACE complexes with those obtained by AlphaFold 3 as well as with the crystal structure of the HSA and the neonatal Fc receptor (FcRn) complex. It was found that domains DI and DIII of albumin are involved in binding both ACE and FcRn. The obtained results of molecular modeling outline the direction for further study of the mechanisms of HSA–ACE interaction in vitro. Information about these mechanisms will help in the design and improvement of pharmacotherapy aimed at modulation of the physiological activity of ACE.

## 1. Introduction

Angiotensin I-converting enzyme (ACE) inhibitors are recommended as first-line drugs for antihypertensive therapy, especially in patients with diabetes mellitus and cardiovascular diseases [1]. There are cases reported in which infusion of human serum albumin (HSA) evokes paradoxical hypotension in surgery patients [2,3,4]. A connection was revealed between the development of hypotension and the use of ACE inhibitors by these patients [4]. ACE is a Zn-dependent proteinase that catalyzes the cleavage of angiotensin I decapeptide to angiotensin II octapeptide in the intercellular space and, thus, plays a key role in the renin–angiotensin–aldosterone system (RAAS), which is one of the hormonal systems in humans and mammals responsible for the regulation of arterial blood pressure. In 2012 (the print version of this article was published only in 2014), another study was presented on the connection between HSA and ACE: a large-scale screening of residents of the Japanese islands found that a low serum albumin level was a significant predictor of the development of hypertension [5]. Thus, a sufficient amount of data has accumulated indicating the participation of albumin in the RAAS.

Evidence that albumin could directly interact with ACE was first published in 1979 [6]. Klauser et al. showed in vitro with purified human lung and kidney ACE and commercial HSA that albumin inhibited ACE activity in a noncompetitive manner. Rediscovery of the inhibitory activity of albumin towards ACE occurred in 2014 [7]. Fagyas et al., crediting the authors of [6] as pioneers, have expanded the understanding of the interaction of HSA and ACE. In particular, it was found that HSA has a higher affinity for the C-terminal active site [7].

Over the past few years, the group of Dr. Danilov has been running a project to phenotype some possible mutations in the ACE sequence [8,9,10,11]; as part of this ongoing project, it is of particular interest to analyze whether these mutations might affect the HSA–ACE interaction. One of the recent studies [11] described a patient with a unique ACE phenotype, the feature of which was a reduced ability of albumin to inhibit ACE activity. In this particular case, this feature appeared to be a consequence of competition between HSA and another protein (presumably chemokine CCL18) for binding to ACE due to a high level of CCL18 in this patient. However, the authors suggested that changes in the local conformation of ACE near the cleft between the N- and C-domains, caused by certain mutations in the amino acid sequence of the enzyme, could be of pathophysiological (clinical) significance, since carriers of this phenotype would have a weakened inhibitory power of albumin towards ACE. These patients, due to a high concentration of angiotensin II, could be susceptible to various cardiovascular complications.

As for albumin mutations, there are dozens of genetic variants of HSA. In the 1980s and 1990s, papers appeared that reported on how mutations affected the functional properties of albumin. Kragh-Hansen et al. investigated the possible effects of certain point mutations on the ligand-binding ability of HSA during the interaction of five structurally characterized genetic variants of the protein with the high-affinity pharmaceutical drugs warfarin, salicylate and diazepam [12]. Point mutations can affect the ability of albumin to bind metal cations [13], then its thermostability [14] and finally its affinity for bilirubin, prostaglandins, fatty acids (FA) and hormones [15,16].

However, neither the molecular mechanisms of the HSA–ACE interaction nor the effect of mutations or modifications of albumin (oxidation, glycation) on this interaction have been studied, even though the data on the molecular mechanism of the HSA–ACE interaction could facilitate the development of a fundamentally new type of ACE inhibitor. Direct ACE inhibitors have a spectrum of side effects [17], in particular coughing as a result of elevated levels of bradykinin [7]. It was hypothesized that decreased ACE activity in the brain, either due to genetic mutation or the effects of ACE inhibitors, could be a risk factor for Alzheimer’s disease (AD) since ACE is able to convert amyloid Aβ42 to Aβ40 [10]. On the other hand, it was shown that HSA only partially inhibited bradykinin breakdown [7], so it seems that exogenous and endogenous ACE inhibitors differently affect a number of its functions, including the cleavage of Aβ42. Therefore, modulation of the HSA–ACE interaction could be a safer way of inhibiting ACE. On the other hand, information on the molecular mechanisms of HSA binding to ACE will make it possible to improve the therapy for patients with pathologies accompanied by oxidation and/or glycation of HSA as well as for patients carrying genetic mutations in the structure of HSA and/or ACE. Moreover, albumin as a transport protein plays a key role in the pharmacokinetics of various pharmaceuticals, including some ACE inhibitors [18,19], as well as some bioactive compounds modulating development of AD, such as the NMDA receptor blocker memantine [20] or alkaloid Huperzine A [21]. In the context of developing a personalized AD therapy, it is important to know how the mechanisms of the interactions between HSA, ACE and drugs affect each other.

While the structure of the HSA–ACE complex has not been resolved experimentally, some preliminary details about the mechanism of interaction between the proteins can be obtained using in silico tools. The purpose of the presented work is to use computer modeling methods to study the interaction of native albumin with ACE.

## 2. Results

### 2.1. Macromolecular Docking of HSA onto the Surface of ACE

**Figure 1:**
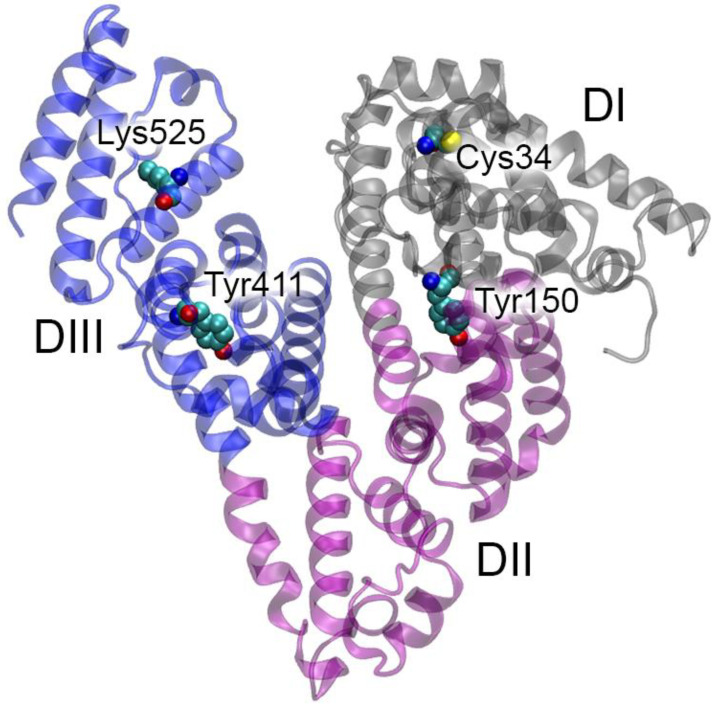
Structural organization of human serum albumin (HSA). HSA domains DI, DII and DIII are represented by a gray, purple and blue ribbon, respectively. The key amino acids of HSA (the redox site Cys34, the main glycation site Lys525 as well as Tyr150 of site Sudlow I and Tyr411 of site Sudlow II, which play a key role in the binding and (pseudo)esterase activity of albumin) are shown as spheres (cyan, blue, red and yellow spheres represent carbon, nitrogen, oxygen and sulfur atoms, respectively). Hydrogen atoms are omitted for clarity.

The three-dimensional structure of HSA (UniProt [22] ID P02768) was first resolved in 1992 [23]. Three homologous domains (DI, DII and DIII), consisting of two subdomains (A, B), form the HSA three-dimensional structure, which is quite labile (Figure 1).

Albumin can bind a wide range of endogenous and exogenous ligands [24]. The protein is a natural cargo for FA [25]. FA can bind in seven main binding sites (FA1-7) [26,27]; two additional FA binding sites (FA8 and FA9) are located in the cleft between domains DI and DIII and can be occupied only in the presence of short-chain FA or saturating amounts of FA, respectively [27]. The binding of exogenous ligands occurs in two main sites: drug site I (site Sudlow I, overlaps with FA7 in subdomain DIIA) and drug site II (site Sudlow II, overlaps with FA3-4 in subdomain DIIIA) [27,28]. Later, drug site III was identified in subdomain DIB, which overlaps with FA1 and binds some compounds such as hemin and bilirubin [29,30]. Some ligands (e.g., thyroxine and lidocaine) bind in the cleft between domains DI and DIII [31,32]. Site Sudlow II with catalytically active Tyr411 (Figure 1) is known to be the main site of pseudoesterase activity of albumin towards esters [33]. Earlier, we substantiated that site Sudlow I with catalytic Tyr150 (Figure 1) can be responsible for true esterase activity of the protein [34].

In addition to non-covalent binding of various ligands, albumin can be subject to covalent modifications. Thus, the albumin molecule contains a free thiol group as part of Cys34 (domain DI, Figure 1), the presence of which determines the redox modifications of albumin: cysteinylation, homocysteinylation and sulfinylation [35]. Another known type of modification of HSA is the covalent binding of sugars at lysines and arginines. Lys525 (domain DIII, Figure 1) is known as the most reactive glycation site of HSA [36].

**Figure 2:**
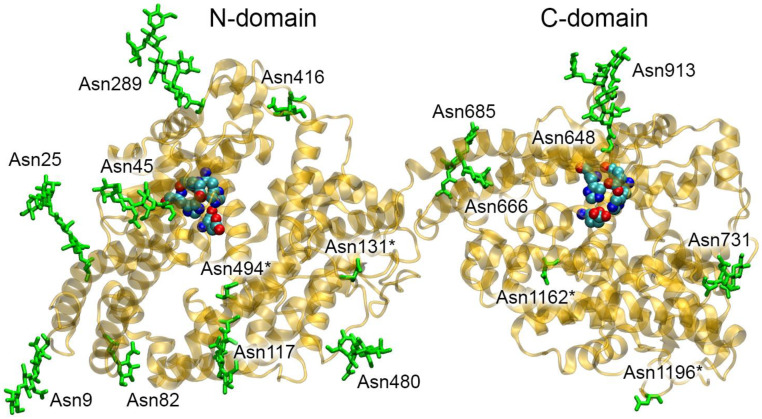
Structural organization of angiotensin I-converting enzyme (ACE). The ACE molecule is represented by a yellow ribbon. Glycosylated ACE residues are shown as green sticks. The asterisk (*) symbols indicate asparagine residues that could potentially be glycosylated in native human ACE and not glycosylated in recombinant ACE (PDB entry 7Q3Y [37]). The active sites of ACE (His361, Glu362, His365 and Glu389 in the N-domain and His959, Glu960, His963 and Glu987 in the C-domain) are shown as spheres (cyan, blue and red spheres represent carbon, nitrogen and oxygen atoms, respectively). Hydrogen atoms are omitted for clarity.

In 2022, the three-dimensional structure of the monomer of recombinant human ACE (UniProt ID P12821) was resolved with the help of cryogenic electron microscopy (PDB entry 7Q3Y [37]). The ACE molecule is a single polypeptide chain containing two highly homologous domains (N- and C-domains, Figure 2). Although both ACE domains contain an active site and a zinc ion, they are not catalytically equivalent. They are distinguished by different rates of peptide hydrolysis, unequal sensitivity to inhibitors and activation profile by chlorine ions [38,39,40,41].

At the first stage, using the GRAMM web server (The University of Kansas, Lawrence, KS, USA, https://gramm.compbio.ku.edu/, accessed on 23 September 2024) [42], molecular docking of an unmodified and fatty acid-free HSA molecule onto the ACE surface was performed. The result of running the GRAMM program was the 10 most probable conformations of HSA–ACE complexes. Table 1 presents the structural characteristics of the resulting complexes: the number of atoms in the HSA and ACE molecules forming close contacts between proteins (at a distance of no more than 3.5 Å), the main amino acids of HSA and ACE involved in the contacts as well as the amino acids involved in specific interactions (hydrogen bonds and salt bridges).

**Figure 3:**
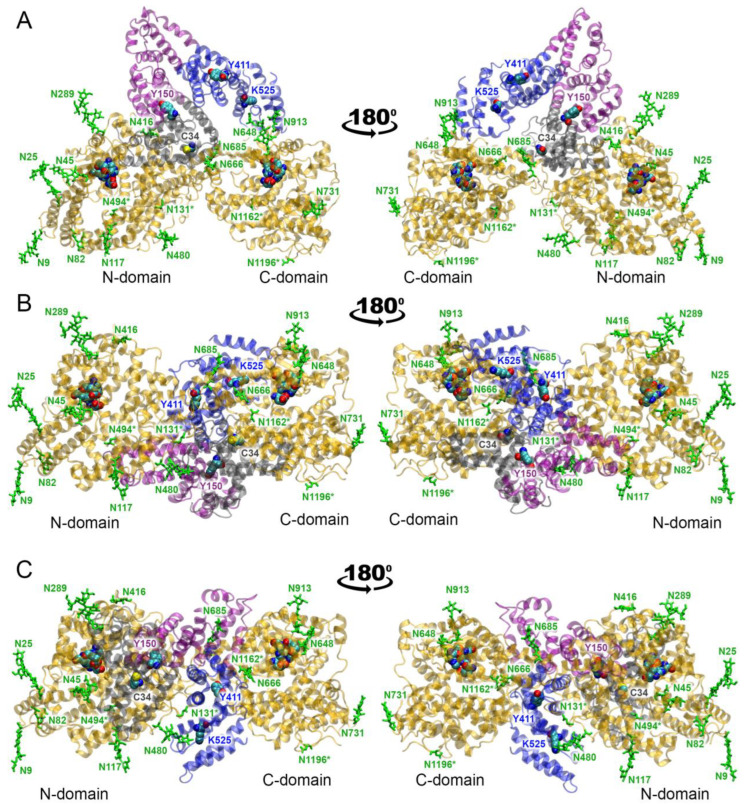
The most probable conformations of the HSA–ACE complex according to macromolecular docking data. (**A**) The leading complex with the maximum number of contacts between HSA and ACE (complex 4). (**B**) The second complex by the number of contacts between HSA and ACE (complex 7). (**C**) The third complex by the number of contacts between HSA and ACE (complex 6). HSA domains DI, DII and DIII are represented by gray, purple and blue ribbons, respectively. The ACE molecule is represented by a yellow ribbon, and the glycosylated ACE residues are shown with green sticks. The asterisk (*) symbols indicate asparagine residues that could potentially be glycosylated in native human ACE and were not glycosylated in the recombinant ACE used for in silico experiments. N- and C-domains of ACE are designated. The key amino acids of HSA and the active sites of ACE are shown as spheres (cyan, blue, red and yellow spheres represent carbon, nitrogen, oxygen and sulfur atoms, respectively). Hydrogen atoms are omitted for clarity.

In computational experiments, it is customary to evaluate the efficiency of the interaction between a ligand and a receptor by the value of free energy of binding Δ*G*. One of the main problems in calculating Δ*G* is that it is very difficult to estimate the entropy component with good accuracy, which is especially critical for macromolecular complexes. Therefore, for protein–protein complexes, it is more rational to estimate the binding strength by the contacts between proteins, their number and type (steric or electrostatic). The more atoms that participate in the formation of steric and polar interactions between proteins, the stronger and more specific the complex. In our work, we used exactly this approach and assessed the strength of the HSA–ACE complexes by the number of atoms forming close contacts between the proteins (at a distance of no more than 3.5 Å). According to this characteristic, complex 4 is the leading one. Complexes 7 and 6 are second and third, respectively. Figure 3A shows the leading complex 4. In this complex, albumin interacts with the glycosylated residues Asn685 and Asn913 of ACE. However, these interactions are exclusively steric; no specific interactions are formed between albumin and sugars.

In complex 7 (the second by the number of contacts between HSA and ACE), the glycosylated residue Asn480 forms steric interactions with the albumin surface; the other glycosylated residues of ACE do not interact with HSA (Figure 3B). In complex 6 (the third by the number of contacts between HSA and ACE), albumin sterically interacts with the glycosylated residues Asn45, Asn117, Asn666 and Asn685 of ACE (Figure 3C). Asn685 additionally forms a hydrogen bond with Ser304 of HSA.

Summarizing the result of macromolecular docking, it can be noted that, in complex 4, domain DI of HSA binds in the cleft between the N- and C-domains of ACE, domain DII of HSA binds to the N-domain of ACE, and domain DIII of HSA binds to the C-domain of ACE (Figure 3A). In complex 7, domains DI and DIII of HSA bind in the cleft between the N- and C-domains of ACE, while domain DII of HSA does not contact the surface of the enzyme (Figure 3B). Finally, in complex 6, domains DII and DIII of HSA bind in the cleft between the N- and C-domains of ACE, and domain DI of HSA contacts the N-domain of the enzyme (Figure 3C). At the next stage, we checked the stability of these structures by molecular dynamics simulation.

### 2.2. Molecular Dynamics of HSA–ACE Complexes

Table 2 summarizes the results of the conformational analysis of complexes 4, 7 and 6 in dynamics: the number of atoms in the HSA and ACE molecules forming close contacts between the proteins; the main amino acids of HSA and ACE participating in these contacts in the final conformation after 100 ns of simulation; amino acids involved in specific interactions and the lifetime of these interactions as a percentage of the total length of the simulation.

For all three complexes, the number of contacts between HSA and ACE decreases in aqueous solution, and most of the specific interactions in the complexes obtained by docking are lost; however, new interactions are formed during the simulation (Table 2). Complex 4 turned out to be the most stable and strong according to MD simulation. The architecture of complex 4 does not change during the simulation: domain DI of HSA remains bound in the cleft between the N- and C-domains of ACE, domain DII of HSA interacts with the N-domain of ACE, and domain DIII of HSA contacts the C-domain of ACE. In this complex, 13 specific interactions persist over 50 ns of the simulation (Table 2). On the contrary, the topologies of complexes 7 and 6 change within 100 ns of simulation. In complex 7, the interaction between domain DIII of HSA and the N-domain of ACE is lost; nevertheless, there are nine specific contacts in this complex that persist for more than 50% of the simulation. In complex 6, the interaction is lost between domain DII of HSA and the C-domain of ACE, and the interaction is lost between domain DIII of HSA and both ACE domains. There are only four long-lived specific interactions between the proteins.

Additionally, we calculated the values of the root means square deviation (RMSD) of Cα-atoms of complexes 4, 7 and 6 (Figure S1). Complex 7 appeared to be the most stable; for this complex, the value of the RMSD stabilized after 40 ns of the simulation. Complex 4, despite the fact of being the strongest, is less stable compared to complex 7. However, its RMSD value stopped rising after 60 ns of the simulation, indicating stabilization of the polypeptide chain. For complex 6, the value of the RMSD increased during the entire simulation, indicating that the initial conformation of this complex obtained by docking is far from being natural.

Thus, the ranking of conformations of the HSA–ACE complex according to macromolecular docking corresponds to the ranking of these conformations according to MD simulation. That is, macromolecular docking makes it possible to obtain a first approximation of the structure of the HSA–ACE complex, but this structure must be refined by the method of molecular dynamics. Based on the MD results, complex 6 appears to be the least probable of the three considered, so we exclude it from further consideration.

### 2.3. How Binding to ACE Can Affect HSA Activity According to Molecular Modeling

**Figure 4:**
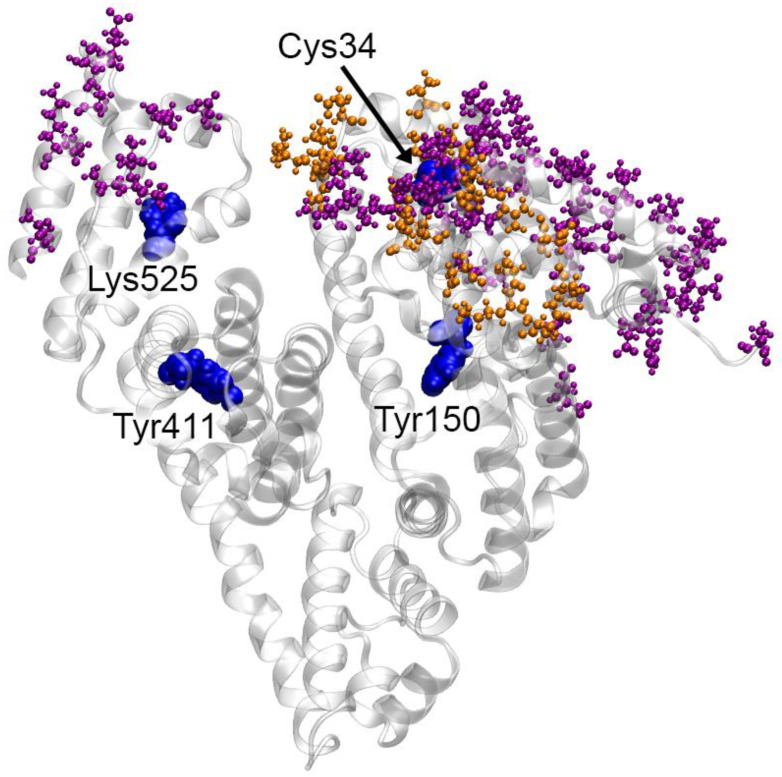
Amino acids on the surface of HSA interacting with ACE in complexes 4 (purple) and 7 (orange) according to molecular dynamics (MD) simulation. The HSA molecule is represented by a gray ribbon. Blue spheres highlight the key amino acids of HSA: the redox site Cys34, the main glycation site Lys525, Tyr150 of site Sudlow I and Tyr411 of site Sudlow II.

In complexes 4 and 7, the main interacting site of HSA is the surface of domain DI; in complex 4, several amino acids of domain DIII are additionally involved in the interaction with ACE (Figure 4).

In both complexes, the maximum contact between HSA and ACE occurs in the region near Cys34 of albumin. Therefore, it can be expected that redox modification of Cys34 will change the features of HSA binding to ACE, and conversely, ACE binding will have the maximum effect on the redox activity of HSA. Thus, in complex 4, Asp38 of HSA interacts with Lys622 of ACE (Table 2), thus blocking access to the thiol group of Cys34. In complex 7, the side chain of Asp38 of HSA forms a salt bridge with Arg797 of ACE (Table 2). Although this interaction does not limit access to the thiol group of Cys34, the redox activity of the latter can still be reduced. This is due to the fact that Asp38 of free HSA along with Tyr84 stabilize the reactive thiolate (deprotonated form of the cysteine) by attracting the proton of its SH group [43]. In complexes with ACE, the carboxyl group of Asp38 interacts with the amino acids of the enzyme, thus reducing the reactivity of Cys34. An opposite effect is also possible: oxidation of albumin at the thiol group of Cys34 can affect the local conformation of domain DI and, as a consequence, affect the affinity of albumin for ACE. Other sites of HSA activity (catalytic tyrosines Tyr150 and Tyr411 in the sites Sudlow I and II as well as the glycation site Lys525) are not directly affected by the interaction with ACE; however, we do not exclude the possibility of allosteric effects. Their analysis requires additional experiments, which will be the task of our future research.

### 2.4. How Binding to HSA Can Affect ACE Activity According to Molecular Modeling

**Figure 5:**
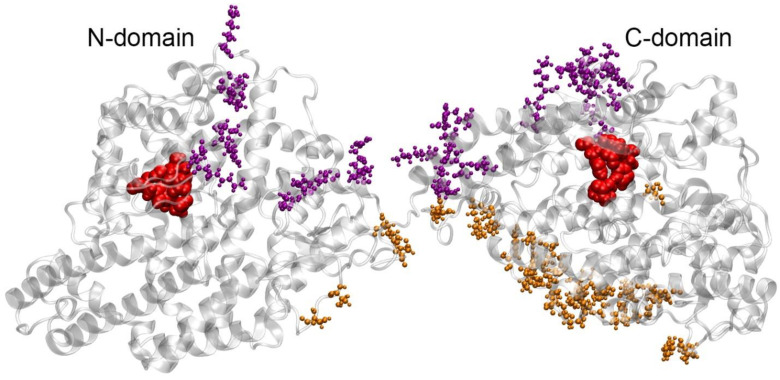
Amino acids on the surface of ACE interacting with HSA in complexes 4 (purple) and 7 (orange) according to MD simulation. The ACE molecule is represented by a gray ribbon. The amino acids of the active sites of ACE are shown in red.

Figure 5 shows the amino acids of ACE interacting with HSA in complexes 4 and 7.

Complex 4, as noted above, is the strongest of those found. As can be observed in Figure 5, several ACE amino acids that contact HSA in this complex flank the entrance to the active site of the N-domain, that is, the albumin molecule closes the entrance to the active site. Thus, we believe that, in complex 4, HSA competitively inhibits the activity of the N-domain of ACE. In complex 7, HSA actually does not interact with the N-domain of ACE and has a large number of contacts with the C-domain (Figure 5). At the same time, albumin does not block the entrance to the active center of the C-domain, which indicates that, in complex 7, allosteric modulation (non-competitive type of inhibition) of the activity of the C-domain is more likely to occur. The topology of complex 7 is more consistent than that of complex 4 with the literature data, according to which, firstly, albumin inhibits ACE activity in a non-competitive manner [6], and secondly, HSA inhibits the activity of the C-domain of ACE to a greater extent than the activity of the N-domain [7]. In these publications, there is no information about the competitive inhibition of ACE, which suggests the dominance of the Cys34-oxidized form of the albumin molecule.

### 2.5. How Point Mutations in HSA and ACE Can Affect Their Interaction According to Molecular Modeling

**Figure 6:**
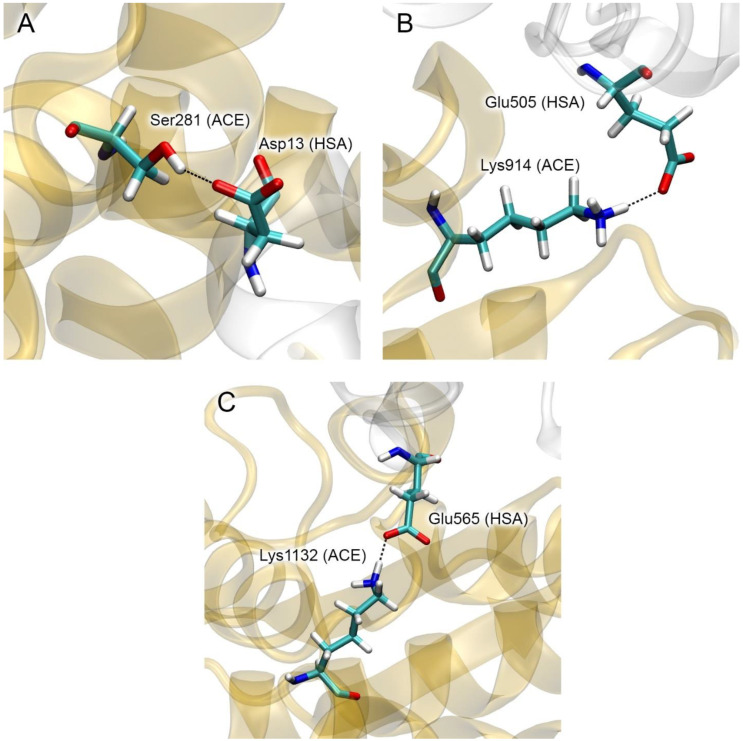
Specific interactions in complex 4 that could potentially be affected by mutations in the albumin molecule: Asp13Asn (**A**), Glu505Lys (**B**) and Glu565Lys (**C**). HSA and ACE molecules are represented by white and yellow ribbons, respectively. The interacting amino acids are shown as sticks (carbon, hydrogen, nitrogen and oxygen atoms are shown in cyan, white, blue and red, respectively).

Among the HSA residues that are involved in the interaction with ACE in complex 4 according to MD (Table 2), mutations Asp13Asn, Glu505Lys and Glu565Lys are known [16,44]. Figure 6 shows the interactions in which these residues are involved.

In the case of the Asp13–Ser281 interaction (Figure 6A), when Asp13 is replaced with Asn, the hydrogen bond between these amino acids is likely to be preserved, and such a replacement will not affect the efficiency of the HSA–ACE interaction. Replacement of the negatively charged Glu505 and Glu565 (Figure 6B,C) with positively charged lysines will have a significant impact on the Glu505–Lys914 and Glu565–Lys1132 interactions, which could hypothetically weaken the strength of the HSA–ACE complex.

**Figure 7:**
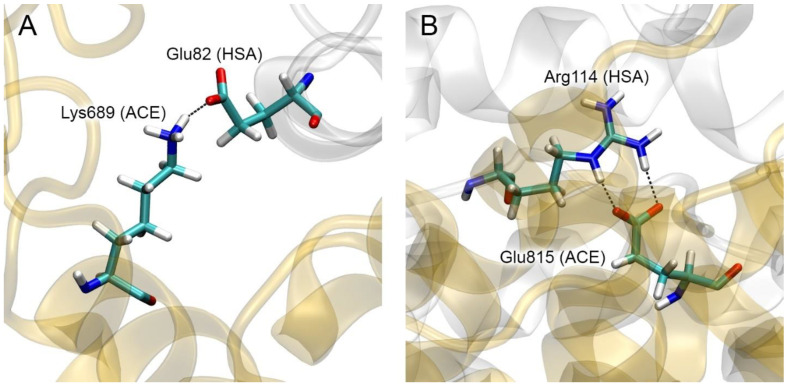
Specific interactions in complex 7 that could potentially be affected by mutations in the albumin molecule: Glu82Lys (**A**) and Arg114Gly (**B**). HSA and ACE molecules are represented by white and yellow ribbons, respectively. The interacting amino acids are shown as sticks (carbon, hydrogen, nitrogen and oxygen atoms are shown in cyan, white, blue and red, respectively).

Among the HSA residues that are involved in the interaction with ACE in complex 7 according to MD (Table 2), mutations Glu82Lys and Arg114Gly are known [16]. Figure 7 shows the interactions in which these residues are involved. Replacement of negatively charged Glu82 (Figure 7A) with a positively charged lysine will have a significant impact on the Glu82–Lys689 interaction, which may weaken the strength of the HSA–ACE complex. Replacement of massive and positively charged Arg114 (Figure 7B) with neutral and much less massive Gly will weaken both the steric and electrostatic interactions between HSA and ACE at this site, which may also weaken the strength of the complex.

As for the reverse situation (that is, when any mutation can lead to increased interaction between HSA and ACE), the amino acid Lys573 in HSA can be noted. In complex 4, this lysine is close to the surface of ACE but does not form any specific interactions (Table 2). The Lys573Glu mutation in the HSA molecule is described in the literature [16]. In complex 4, Lys893 of ACE is located in the vicinity of Lys573 of HSA. Substitution of Lys573 of HSA for glutamate may result in the formation of a salt bridge between this glutamate and Lys893 of ACE, which may enhance the HSA–ACE interaction. In complex 7, we did not identify such “amplifying” mutations.

Regarding ACE, Danilov et al. provide a list of known ACE mutations associated with various diseases [10]. According to the result of MD simulation, none of these amino acids are involved in the interaction with HSA, so these mutations do not appear to affect the HSA–ACE interaction.

### 2.6. HSA–ACE Complexes Acording to AlphaFold 3

In 2024, AlphaFold 3 (AF3) was introduced, which is a new tool for predicting interactions between macromolecules based on deep learning architecture [45]. In addition to the macromolecular docking, we also applied AF3 to construct HSA–ACE complexes. Five possible conformations of the HSA–ACE complex were obtained. The obtained complexes were processed as described in Section 4.4. Three complexes with the highest number of contacts between HSA and ACE were selected for further analysis (complexes 1-AF, 2-AF and 3-AF). The result is shown in Figure S2 and Table S1 in Supplementary materials. In complex 1-AF, domains DI and DIII of HSA interact with the C-domain of ACE, and domain DII of HSA binds in the cleft between the N- and C-domains of ACE (Figure S2A). In complex 2-AF, domain DI of HSA interacts with the N-domain of ACE, domain DII of HSA binds in the cleft between the N- and C-domains of ACE, and domain DIII of HSA is not involved in the interaction with ACE (Figure S2B). In complex 3-AF, domains DI and DII of HSA interact with the N-domain of ACE, while domain DII of HSA and the C-domain of ACE are not involved in the interaction (Figure S2C).

Interestingly, the interaction between HSA and ACE according to AF3 (Table S1) is much weaker than according to GRAMM (Table 1). The HSA–ACE complex has not yet been resolved experimentally so far. However, the structure of albumin with another macromolecular structure is known, namely with the neonatal Fc receptor (neonatal fragment crystallizable receptor, FcRn). FcRn is an intracellular receptor, which is necessary for delivery of newly synthesized albumin to the basolateral side of the cells and subsequent secretion of albumin into the bloodstream. The mechanism of interaction of albumin with FcRn is well studied: in 2014, the structure of their complex was obtained by X-ray diffraction (PDB entry 4N0F [46]). We processed structure 4N0F in the same way as the HSA–ACE complexes (adding of hydrogen atoms and optimization by the energy minimization method). In the resulting HSA–FcRn complex, we found 13 specific contacts (hydrogen bonds and salt bridges) between the proteins, although the size of the FcRn molecule is four-fold smaller than the size of ACE. AF3 is a powerful tool for predicting biomolecular interactions, so we do not exclude that the interaction between HSA and ACE is indeed weaker compared to FcRn. We also do not exclude that the optimization of the structure of AF3 complexes of HSA–ACE by MD simulation may reveal additional details of the interaction between these proteins. This is the task of our future studies.

### 2.7. Limitations of the Research

The main limitations of the presented work are related to the imperfection of the scoring functions of the programs for macromolecular docking. We do not exclude that the real conformation of the HSA–ACE complex is not the most probable according to the scoring function of GRAMM. AF3 is a new powerful tool for predicting interactions between macromolecules. However, the advantage of GRAMM over AF3 is that experimentally obtained protein structures are used for the docking procedure. Thus, we suppose that, at present, there is no perfect program that can predict the structure of the complex between two large proteins, such as HSA and ACE, with accuracy comparable to the experimental procedures, especially taking into account the susceptibility of albumin to allosteric modulation and extensive glycosylation of ACE.

**Figure 8:**
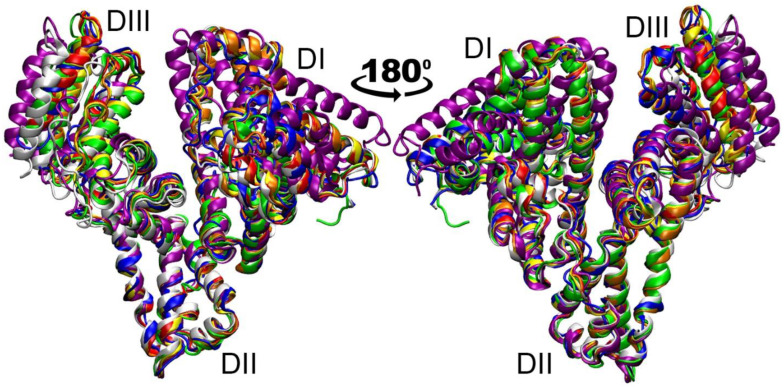
Comparative analysis of albumin structures with different ligand loading: free HSA (blue); HSA with a warfarin molecule in site Sudlow I (red), HSA with a diazepam molecule in site Sudlow II (orange), HSA with a 4Z,15E-bilirubin-IX-alpha molecule in site III (yellow), HSA with a lidocaine molecule in the cleft between domains DI and DIII (green, used in the presented work), HSA with seven oleic acid molecules in FA binding sites (purple) and HSA in complex with FcRn (white).

Another limitation of the results obtained is that we used only one albumin structure at this stage of the study. It was chosen because it is free of mutations and contains a minimal number of unresolved terminal amino acids compared to the other structures available in the PDB database. In addition, we considered it necessary to use FA-free albumin at the first stage for the reason that the total concentration of major FA in a healthy body does not exceed 100 µM; therefore, part of the albumin molecules (the concentration of which in plasma is 500–700 µM) is free of FA. However, it is known that albumin is susceptible to allosteric modulation, and binding of various ligands can affect its structure, which in turn can affect the results of computer simulation. Figure 8 shows a superposition of different HSA structures from the PDB: free HSA (1UOR [23], blue), HSA with a warfarin molecule in site Sudlow I (2BXD [47], red), HSA with a diazepam molecule in site Sudlow II (2BXF [47], orange), HSA with a 4Z,15E-bilirubin-IX-alpha molecule in site III (2VUE [48], yellow), HSA with a lidocaine molecule in the cleft between domains DI and DIII (3JQZ [32], green, used for modeling in the presented work), HSA with seven oleic acid molecules in the FA binding sites (1GNI [26], purple) and HSA in a complex with FcRn (4N0F [46], white).

As can be observed in Figure 8, the most negligible difference in the structure of albumin is observed in domain DII, and the most significant is observed in domains DI and DIII. In domain DI, structure 1GNI (HSA with seven oleic acid molecules, purple) differs most from the other structures. In domain DIII, the most noticeable differences are observed for structures 1GNI and 4N0F (HSA in complex with FcRn, white). The differences between ligand-free albumin and albumin complexed with low-weight pharmaceuticals (including the structure of HSA with lidocaine that we used in the presented work) are much less pronounced. Therefore, in future studies, it seems important to perform additional computational experiments using structures 1GNI and 4N0F as models of albumin.

## 3. Discussion

As noted above, it has been confirmed that albumin is an endogenous inhibitor of ACE [7]; however, little is known about the mechanism of interaction between these proteins. There are no experimental data on which amino acids of HSA and ACE are involved in binding or on how the binding efficiency changes when these proteins are modified. In the presented work, using macromolecular docking and the molecular dynamics methodology, we presented models of the HSA–ACE complex, though it is not yet possible to assess the realism of the structures we obtained. Recently, molecular docking of the HSA molecule onto the surface of ACE was performed [11]. In the complex obtained by the authors, domain DI of HSA binds between the N- and C-domains of ACE, domain DII of HSA interacts with the C-domain of ACE, and domain DIII of HSA does not interact with the surface of the enzyme. None of the HSA–ACE complexes that we obtained in our work are similar to the structure from the work of Enyedi et al. We believe this may be due to the fact that the authors used an albumin structure loaded with seven palmitic acid molecules for molecular docking, while in our studies, we used albumin free of FA. As mentioned above, binding to FA significantly influences the conformation of HSA (Figure 8) and, as a consequence, may affect its interaction with ACE. In the next steps, it will of course be important to study more thoroughly—with MD application—the interaction of ACE with FA-loaded HSA as well as with oxidized and glycated HSA to study how modifications of albumin affect its interaction with ACE.

**Figure 9:**
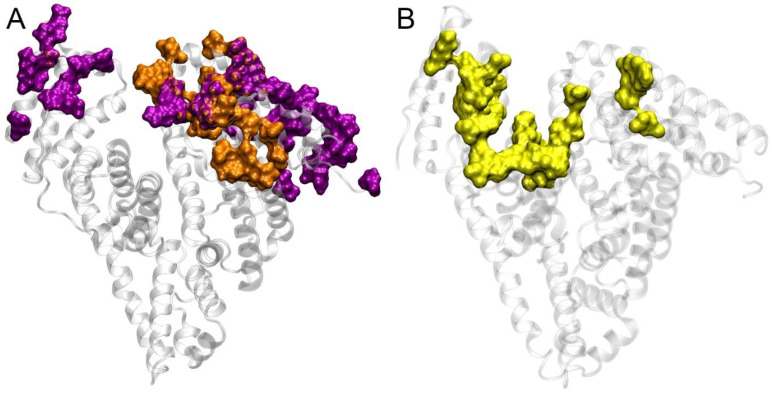
Topology of the sites of interaction of HSA with macromolecules. The surface of HSA is shown in gray, and the surfaces of the sites are shown as colored spheres. (**A**) The sites of interaction of native HSA with ACE according to MD simulation; purple spheres indicate HSA amino acids interacting with ACE in complex 4, orange—in complex 7. (**B**) The site of interaction (shown in yellow) of native HSA with FcRn according to X-ray [46].

In the absence of experimental data on the mechanism of the HSA–ACE interaction, it is still possible to compare our results with known experimental data on the interaction of albumin with FcRn. Figure 9 shows a comparison of the topologies of the possible HSA–ACE complexes that we obtained (complexes 4 and 7) and the experimental structure of the HSA–FcRn complex.

As can be observed in Figure 9, the interaction of HSA with ACE and FcRn involves similar areas on the albumin surface. Of course, these regions are not completely identical; however, the scales of ACE and FcRn also differ significantly: the length of the ACE polypeptide chain in structure 7Q3Y is 1201 amino acids, but the length of FcRn in structure 4N0F is 267 amino acids. However, domains DI and DIII of albumin in both cases are involved in the interaction. It should be noted that, in two of the three strongest HSA–ACE complexes obtained by AF3 (complexes 1-AF and 3-AF), domains DI and DIII of HSA are also the main participants in the interaction with ACE (Figure S2A,C and Table S1).

The role of domains DI and DIII in the physiological activity of albumin has been noted by many researchers. Thus, Paar et al. studied the structural features of albumin in patients with chronic liver disease [49]. The albumin of these patients is oxidized and overloaded with FA and bilirubin. The authors demonstrated that albumin from healthy people has greater flexibility and mobility of domains DI and DIII. Fujiwara et al. showed, with the help of MD simulation, that the binding of myristate to HSA causes the movement of domains DI and DIII, which increases the gyration radius of the protein molecule [50]. Ketrat et al. applied MD simulation to reveal the structural and dynamic properties of canine serum albumin and compared them with the properties of BSA and HSA. It was shown that it is the dynamics of domains DI and DIII that determine the characteristics of each albumin [51].

Above, we suggested that oxidation of HSA at the thiol group of Cys34 may affect its interaction with ACE. The hypothesis is based on the fact that Cys34 oxidation reduces the affinity of HSA for FcRn [52]. It is interesting to note that, according to known experimental data, glycation of Lys525 also weakens the interaction of HSA with FcRn [52,53], although Lys525 does not directly contact FcRn in the crystal structure of the HSA–FcRn complex. That is, an allosteric effect of glycation on the affinity for the receptor takes place. Therefore, despite the fact that there is no direct contact between Lys525 and the surface of the enzyme, we do not exclude the same allosteric effect of glycation for the HSA–ACE interaction.

As for mutations in the albumin molecule, it is difficult to imagine that a point mutation can significantly affect the interaction of two massive proteins with a large interaction area. However, it is known that mutations Glu82Lys and Arg114Gly change the characteristics of the interaction of albumin with FcRn: both mutations increase the value of the dissociation constant (*K*_D_) of the complex by approximately two-fold [54]. Without a doubt, such susceptibility is due to the fact that the three-dimensional structure of albumin has a fairly high conformational lability, and the phenomena of cooperativity and allosteric modulation can occur in its molecule like in multimeric proteins [55,56]. Therefore, we believe that mutations in the albumin molecule can affect the efficiency of the HSA–ACE interaction and, as a consequence, the health of carriers of these mutations. No one has yet conducted research in this area; interesting discoveries are yet to come.

## 4. Materials and Methods

### 4.1. Building of Three-Dimensional Models

As a three-dimensional model of HSA, we used the crystal structure of the protein from PDB entry 3JQZ, chain A [32]. As a three-dimensional model of ACE, we used the structure of the full-length protein monomer obtained by cryogenic electron microscopy, PDB entry 7Q3Y [37]. The molecules of the ligand and unused chain were removed from structure 3JQZ. In structure 7Q3Y, sugar molecules, zinc and sodium ions as well as the molecule of water in the active center were kept. The missing atoms were built using the program Visual Molecular Dynamics v.1.9.4a53 (VMD, University of Illinois Urbana-Champaign, Champaign, IL, USA) [57].

The protonation state of HSA histidines was determined automatically by the software GROMACS 2022.2 (University of Groningen, Groningen, The Netherlands) [58] based on the amino acid environment and a pH = 7.0. Thus, His288 was specified in the HID-form (atom Nδ of the imidazole ring is protonated, and atom Nε is deprotonated), and the remaining histidines of HSA were specified in the HIE-form (atom Nδ of the imidazole ring is deprotonated, and atom Nε is protonated). In ACE structure 7Q3Y, the active site of the N-domain contains a zinc ion and a water molecule, so catalytic His361 and His365 were manually assigned in the HID-form according to the reaction mechanism [59]. The active site of the C-domain in structure 7Q3Y is empty, so the protonation state of catalytic His959 and His963 was automatically determined by GROMACS as the HIE-form. The protonation state of the other histidines of ACE was also determined automatically: HID-form for His164, His958, His986 and His1018 and HIE-form for the other histidines.

### 4.2. Macromolecular Docking

The prepared HSA and ACE models were used for the macromolecular (protein–protein) docking procedure. Docking was performed using the GRAMM web server (The University of Kansas, Lawrence, KS, USA, https://gramm.compbio.ku.edu/, accessed on 23 September 2024) [42]. The ACE molecule was specified as a receptor, and the HSA molecule was specified as a ligand. The structure of ten resulting complexes was optimized by the energy minimization method in the CHARMM27 force field [60] using the software GROMACS 2022.2 (University of Groningen, The Netherlands) [58], and then, the docking results were analyzed.

### 4.3. Molecular Dynamics

Conformational changes in HSA–ACE complexes were studies by single trajectory MD simulation using the software GROMACS 2022.2 (University of Groningen, The Netherlands) [58] in CHARMM27 force field [60]. Each complex was virtually placed in a cubic periodic box filled with water molecules. The TIP3P water models (transferable intermolecular potential with 3 points) were used to describe water molecules [61]. To neutralize the systems, 44 sodium ions were added (which corresponds to a concentration of approximately 0.012 M). Temperature (300 K) and pressure (1 bar) were kept constant using the V-rescale thermostat [62] and Parrinello–Rahman barostat [63], with coupling constants of 0.1 ps and 2.0 ps, respectively. Long-range electrostatic interactions were treated by the particle-mesh Ewald method [64]. Lennard–Jones interactions were calculated with a cutoff of 1.0 nm. The LINCS algorithm (linear constraint solver for molecular simulations) was used to constrain bond length [65]. Before running the MD simulations, all the structures were minimized by steepest descent energy minimization and equilibrated under NVT (1000 ps) and NPT (5000 ps) ensembles. The timestep for MD simulation was 0.002 ps. The length of the simulation was 100 ns.

### 4.4. Constructing HSA–ACE Complexes Using AlphaFold 3

The primary amino acid sequences of HSA and ACE were inputted into the AlphaFold Server [45]. Glycan chains were added to the ACE sequence according to the PDB structure 7Q3Y. The run of AF3 resulted in five possible models of the HSA–ACE complex. For each model, hydrogen atoms were built using the program VMD v.1.9.4a53 (University of Illinois Urbana-Champaign, USA) [57]. Then, the structure of the resulting complexes was optimized by the energy minimization method in the CHARMM27 force field [60] using the software GROMACS 2022.2 (University of Groningen, The Netherlands) [58].

## 5. Conclusions

In the presented work, we studied the interaction of HSA with ACE using computer modeling methods to obtain preliminary details about the mechanism of their interaction. Using the macromolecular docking method and subsequent molecular dynamics simulation, we obtained the two most probable conformations of the HSA–ACE complex. Analysis of these conformations showed that the processes of oxidation of Cys34 of HSA and the binding of HSA to ACE can reciprocally influence each other. Known point mutations in the HSA molecule, Glu82Lys, Arg114Gly, Glu505Lys, Glu565Lys and Lys573Glu, may also affect the interaction of albumin with ACE. These two assumptions are justified by the fact that, according to known experimental data, the oxidation of Cys34 of HSA as well as some point mutations in the albumin molecule affect the efficiency of its interaction with another macromolecule, namely FcRn. According to the result of MD simulation, known ACE mutations associated with various diseases do not affect the HSA–ACE interaction. A comparative analysis of the resulting HSA–ACE complexes with the known crystal structure of the HSA–FcRn complex showed that the binding both to ACE and to FcRn involves domains DI and DIII of albumin, the role of which in the physiological activity of HSA has been noted by many researchers. The obtained results of molecular modeling outline the direction for further study of the mechanisms of the HSA–ACE interaction in vitro. Information about these mechanisms will help in the design and improvement of pharmacotherapy aimed at modulating the physiological activity of ACE.

## Figures and Tables

**Table 1:** Structural characteristics of HSA–ACE complexes obtained by macromolecular docking: quantity of atoms in HSA and ACE molecules forming close contacts between the proteins (at a distance of no more than 3.5 Å), the main amino acids of HSA and ACE involved in these contacts as well as the amino acids involved in specific interactions (hydrogen bonds and salt bridges).

Complex # Quantity of Interacting Atoms, HSA/ACE	HSA	ACE	Specific Interactions HSA–ACE
1 372/396	Ala172, Asp173, Lys174, Ala176, Pro180, Lys181, Asp183, Glu184, Asp187, Phe228, Lys274, Leu275, Lys276, Glu277, Cys278, Glu294, Asp296, Glu297, Ala300, Asp301, Pro303, Ala306, Ala307, Asp308, Glu311, Asp314, Lys317, Asn318, Met329, Tyr332, Arg336, Pro339, Glu393, Glu396, Gln397, Leu398, Gly399, Glu400, Lys402, Lys439, His440, Pro441, Lys444, Arg445, Lys519	Ala232, Arg235, Met267, Val269, Phe271, Pro272, Asp273, **Asn416**, Asp417, Thr418, Gln579, Trp580, Gln584, Gln587, Glu617, Ala618, Ser621, Lys622, Glu626, Val633, Glu637, Glu640, Asn644, **Asn648**, Ile656, **Asn685**, Ile688, Leu905, Pro906, Pro909, Lys914, Asn937, Lys939, Gln969, Lys971, Asp972, Lys1132, Lys1143, Leu1144	Lys174-**Asn648** (HB) Lys181-Glu637 (SB) Asp187-Lys939 (SB) Lys274-Ala618^O^ (HB) Glu294-**Asn685** (HB) Pro303^O^-Gln584 (HB) Glu311-Gln587 (SB) Lys317-Asp417 (SB) Tyr332-Asp273 (SB) Pro339^O^-**Asn685** (HB) Glu396-Gln969 (HB) Glu396-Lys1143 (SB) Gln397-Lys1132 (HB) Lys439-Asp972 (SB)
2 319/287	Phe11, Lys12, Asp13, Gly15, Glu16, Glu17, Asn18, Glu48, Lys51, Val54, Ala55, Asp56, Glu57, Lys159, Glu167, Ala171, Ala172, Asp173, Ala175, Cys177, Pro180, Lys181, Glu184, Pro282, Leu284, Glu294, Glu297, Glu396, Gln397, Gly399, Glu400, Tyr401, Lys402, Ser435, Lys436, Cys437, Cys438, Lys439, His440, Pro441, Lys444, Arg445	Arg245, Arg453, Arg458, Phe461, Tyr465, Lys469, Val477, Thr478, **Asn480**, Gly589, Val591, Trp594, Pro595, Glu596, Tyr597, His600, Leu603, Asp605, Pro608, Glu609, Pro803, Ser804, Gln807, Glu810, Arg811, Gln814, Glu815, Gln817, Leu821, Lys1067, **Asn1162**, Leu1190	Ala172^HN^-Glu609 (HB) Asp173-**Asn480** (HB) Glu294-Arg245 (SB) Glu396-Tyr465 (HB) Glu396-Lys469 (SB)
3 278/267	Ala2, His3, His67, Pro96, Glu208, Lys212, Ala229, Ser232, Lys233, Thr236, Thr239, Lys240, Thr243, His247, Ala320, Lys323, Asp324, Val325, Thr355, Glu358, Lys359, Cys361, Ala362, Ala363, Pro366, Glu376, Pro379, Asp471, Arg472, Lys475, Glu479, Asn483, Ala490, Glu492, Glu495, Thr496, Lys538	Glu212, Gln216, Glu219, Arg235, Arg245, Arg458, Gln586, Gln587, Asn588, Gly589, Glu590, Val591, Glu596, Tyr597, Gln598, **Asn685**, Thr686, Thr687, Arg811, Gln814, Glu815, Pro818, Asp972, Leu973, Arg1137, Leu1144, Arg1148, Leu1156, Gln1160, Pro1161, **Asn1162**, Ser1166	Glu208-Arg245 (SB) Lys212-Gly589^O^ (HB) Ser232-Gln587^O^ (HB) Thr236-Glu590^O^ (HB) Asp324-Arg235 (SB) Glu358-Thr686 (HB) Cys361^O^-**Asn685** (HB) Asp471-Arg811 (SB) Arg472-Glu815 (SB)
4 540/543	His3, Ser5, His9, Lys12, Asp13, Leu14, Gly15, Glu16, Glu17, Asn18, Lys20, Gln33, Cys34, Pro35, Phe36, Glu37, Asp38, His39, Lys41, Asn44, Glu45, Glu48, Lys51, Thr52, Val54, Glu57, Thr79, Leu80, Glu82, Thr83, Tyr84, Asn111, Pro113, Val116, His128, Asp129, Asn130, Glu131, Glu132, Leu135, Leu155, Ala158, Lys159, Lys162, Thr166, Asp259, Lys262, Cys265, Glu266, Cys279, Glu280, Lys281, Pro282, Leu283, Leu284, Lys286, Glu505, Thr508, His510, Ala511, Asp512, Asp563, Lys564, Glu565, Thr566	Asn145, Ala148, Ser149, Arg151, Arg231, Ala232, Arg235, Arg236, Asn263, Tyr265, Asp266, Met267, Val268, Val269, Phe271, Pro272, Asp273, Lys274, Pro275, Asn276, Leu277, Asp278, Thr280, Ser281, Thr282, Leu284, Cys330, Arg350, Thr352, Leu410, Leu411, Asp412, Arg413, Thr415, Asp417, Glu419, Asn423, Lys427, Asn588, Asp616, Glu617, Ala618, Glu619, Ser621, Lys622, Phe623, Glu625, Glu626, Arg629, Lys670, Tyr671, Gln674, **Asn685**, Lys893, Asp896, Thr900, Pro906, Pro909, **Asn913**, Lys939, Lys971, Lys1132	Asp13-Ser281 (HB) Glu16-Lys427 (SB) Glu17-Tyr265 (HB) Glu17-Asn423 (HB) Asn18-Asn276^O, NH^ (HB) Lys20-Asp266 (SB) Gln33-Glu625 (HB) Asp38-Glu619^HN^ (HB) Asp38-Ala618^HN^ (HB) Lys41-Asp616 (SB) Lys51-Asn263 (HB) Glu57^O^-Arg350 (HB) Thr79-Glu619 (HB) Glu82-Gln674 (HB) Thr83-Lys622 (HB) Tyr84-Lys622 (HB) Asn111-Glu625 (HB) His128^O^-Asn588 (HB) Asp129-Arg235 (SB) Asn130-Met267^O^ (HB) Glu131-Phe271^HN^ (HB) Lys159-Glu419 (SB) Lys159-Asp273^O^(HB) Lys162-Glu419 (SB) Lys262-Leu410^O^ (HB) Glu280-Arg413 (SB) Lys286-Asp412 (SB) Glu505-Lys939 (SB) Asp512-Lys971 (SB) Asp563-Lys1132 (SB) Glu565-Thr900 (HB)
5 347/327	Ala2, His3, Ala8, Phe11, Lys12, Cys53, Val54, Ala55, Asp56, Ser58, His128, Asp129, Phe156, Arg160, Ala163, Ala164, Thr166, Glu167, Cys168, Gln170, Ala172, Asp173, Leu178, Pro180, Lys181, Asp183, Glu184, Asp187, Glu188, Glu266, Asn267, Lys274, Glu277, Lys281, Pro282, His288, Cys289, Glu292, Glu294, Glu297, Cys438, Lys439, His440, Glu442	**Asn131**, Ala134, Arg151, Arg236, Pro272, Asp273, Leu284, Gln285, Glu320, Lys321, Ala323, Arg350, Thr352, Glu609, Asp612, Leu613, Val614, Asp616, Glu617, Ala618, Glu619, Lys622, Arg629, Thr630, Gln632, Val633, Lys670, Thr673, Gln674, Lys677, Phe678, **Asn685**, Leu786, Lys939	Cys53^O^-Arg350 (HB) His128^O^-Ala134^HN^ (HB) Arg160-Glu619 (SB) Ala164^O^-Lys677 (HB) Gln170-Glu609^O^ (HB) Leu178^O^-Lys677 (HB) Glu188-Lys622 (SB) Glu294-**Asn685** (HB) Glu297- **Asn685** (HB) Lys439-Arg629^O^ (HB) Glu442-Lys939 (SB)
6 472/432	Asp13, Glu16, Glu17, His128, Glu131, Lys159, Lys162, Thr166, Ala172, Glu184, Ala258, Asp259, Lys262, Glu266, Ser273, Lys274, Lys276, Glu277, Lys281, Pro282, Leu283, Leu284, Glu294, Glu297, Met298, Pro299, Leu302, Ser304, Leu305, Ala306, Tyr334, Arg337, His338, Pro339, Asp340, Tyr341, Lys372, Phe374, Asp375, Phe377, Lys378, Pro379, Val381, Glu382, Glu383, Gln385, Lys389, Lys439, Pro441, Glu442, Ala443, Lys444, Met446	**Asn45**, Ile46, Thr47, Ala48, Glu49, **Asn117**, **Asn131**, Ala134, Thr135, Pro141, Thr144, Asn145, Ile146, Ala148, Ser149, Arg151, Arg236, Asn263, Asp266, Asn276, Thr280, Leu284, Lys321, Ala323, Asp324, Gly325, Arg326, Glu327, Cys330, Arg350, Asp612, Leu613, Val614, Asp616, Ala618, Glu619, Ser621, Lys622, Phe623, Glu625, Glu626, Arg629, Thr630, Ser631, Val633, Val634, Asn636, **Asn666**, His667, Leu669, Lys670, Tyr671, Thr673, Gln674, Lys677, **Asn685**	Glu17^HN^-Ala323^O^ (HB) Glu184-Thr135 (HB) Lys276-Asn145 (SB) Glu277-Thr144 (HB) Glu297-Arg236 (SB) Ser304-**Asn685** (HB) Ala306^HN^-Glu625 (HB) Tyr334-Glu626 (HB) Tyr334-Arg629 (HB) Asp340^HN^-Glu619 (HB) Tyr341-Glu626 (HB) Lys372-Asn636 (SB) Phe377^O^-Lys670 (HB) Gln385-Lys677 (HB)
7 447/444	Gln33, Cys34, Pro35, Phe36, Glu37, Lys41, Val77, Thr79, Leu80, Arg81, Glu82, Thr83, Gly85, Glu86, Met87, Asp89, Cys90, Lys93, Gln94, Glu97, Glu100, Gln104, Asn111, Pro113, Leu115, Val116, Pro118, Glu119, Asp121, Val122, Thr125, Ala126, Asn130, Thr133, Tyr140, Glu141, Arg144, Lys205, His464, Glu465, Pro468, Ser470, Asp471, Thr474, Thr478, His510, Ala511, Asp512, Thr515, Leu516, Glu565, Thr566	Arg240, Tyr241, Leu244, Arg245, Val477, Thr478, **Asn480**, Gly589, Val591, Pro595, Glu596, Trp599, His600, Pro602, Leu603, Asp605, Asn681, Gln682, Lys693, Tyr789, Val790, Asp794, Ser798, Pro803, Ser804, Glu806, Gln807, Asp808, Glu810, Arg811, Gln814, Glu815, Pro818, Leu821, Gln1042, Asp1049, Lys1067, Gln1115, Ala1116, Ser1131, Lys1132, Glu1133, Arg1137, **Asn1162**, Ser1164, Ser1166, Leu1169, Lys1173, Arg1180	Cys34^O^-Gln807 (HB) Lys41-Asp1049 (SB) Thr79-Asp794 (HB) Arg81-Asp605 (SB) Glu82-Ser798 (HB) Glu82^O^-Lys693 (HB) Glu86-Tyr241 (HB) Glu86-Asn681 (HB) Glu86-Gln682 (HB) Glu97-Thr478^HN^ (HB) Gln104-His600^O^ (HB) Glu119-Lys1173 (SB) Glu141-Arg811 (SB) Lys205-Glu596 (SB) Ala511^HN^-Glu1133 (HB) Asp512-Arg1137 (SB) Glu565-Lys1132 (SB)
8 362/390	Gln33, Thr83, Asn111, Leu112, Pro113, Val116, Arg117, Pro118, Glu119, Asp121, Val122, Glu167, Gln170, Ala171, Ala172, Ala176, Cys177, Pro180, Lys181, Glu184, Asp187, Glu277, Lys281, Pro421, Lys432, Lys439, Asn503, Ala504, Glu505, His510, Asp512, Cys514, Thr515, Leu516, Ser517, Lys519, Arg521, Lys524, Glu556, Cys559, Lys560, Lys573	Asn145, Ala148, Ser149, Arg231, His234, Arg235, Gly238, Leu244, Glu262, Asn263, Asp266, Pro270, Pro272, Asp273, Pro275, Asn276, Asp278, Thr280, Ser281, Leu284, Gln285, Arg350, Val351, Thr352, Asp354, Gln355, Asp417, Lys427, Asn588, Gly589, Glu590, Val591, Ala618, Glu619, Ser621, Lys622, Glu625, Arg629, Thr630, Lys670, Gln684, **Asn685**, Thr686, Thr1140, Ser1147, Arg1148, Gln1155, Leu1156	Gln33-Lys622 (HB) Gln33-Glu625 (HB) Arg117-Asp266 (SB) Glu119-Ser149 (HB) Glu167-Thr280 (HB) Gln170-Arg350 (HB) Lys181-Thr280 (HB) Lys281-Gln285 (HB) Lys432-Asp273 (SB) Lys439-Asp417 (SB) Glu505-Ser1147 (HB) Lys524-**Asn685** (HB) Cys559^O^-Arg231 (HB) Lys560-Glu590 (SB) Lys560-Leu244^O^ (HB) Lys573-Gln1155 (HB)
9 351/352	Gln33, Glu82, Thr83, Asn111, Pro113, Arg117, Glu119, Asp121, Val122, Glu167, Gln170, Ala172, Lys174, Pro180, Lys181, Glu184, Asp187, Lys190, Glu277, Lys281, Phe395, Glu400, Tyr401, Lys402, Lys432, Ser435, Lys436, Lys439, His440, Ala504, Glu505, His510, Asp512, Ile513, Cys514, Thr515, Leu516, Ser517, Glu518, Lys519, Arg521, Lys524, Lys525, Glu556, Cys559, Lys560, Asp562, Asp563	Asn145, Ala148, Ser149, Arg151, Phe228, Arg230, Arg231, His234, Arg235, Gly238, Asp239, Asn243, Leu244, Arg245, Glu262, Asp266, Val269, Phe271, Pro272, Asp273, Pro275, Asn276, Asp278, Thr280, Ser281, Gln285, His331, Arg350, Thr352, Thr415, Asp417, Thr418, Glu419, Lys427, Glu583, Gln587, Asn588, Gly589, Glu590, Val591, Asp616, Ala618, Lys622, Glu625, Arg629, Val633, Gln684, **Asn685**, Thr686, Arg1148, Leu1156	Asn111-**Asn685** (HB) Arg117-Asp266 (SB) Glu119-Arg151 (SB) Asp121-Ser149 (HB) Lys190-Asp273 (SB) Glu277-Gln285 (HB) Lys281-Gln285 (HB) Lys432-Asp273 (SB) Ser435-Glu419 (HB) Lys436-Asp273^O^ (HB) Lys439-Asp417 (SB) Ile513^O^-Arg235 (HB) Lys519-Val269^O^ (HB) Lys560-Glu590 (SB) Lys560-Leu244^O^ (HB) Asp562^O^-Asn243 (HB)
10 267/231	Ala78, Thr79, Arg81, Glu82, Glu86, Cys91, Ala92, Lys93, Pro96, Glu97, Glu100, Leu203, Gln204, Lys205, Phe206, Gly207, Glu208, Arg209, Lys212, Lys240, Thr243, Glu244, Lys317, Ala320, Glu321, Ala322, Lys323, Asp324, Val325, Met329, Glu358, Ala363, Pro366, Lys573	Leu129, **Asn131**, Thr133, Ala134, Thr135, Cys136, Trp137, Ile146, Gln285, **Asn289**, Asp612, Leu613, Glu626, Arg629, Thr630, Val633, Glu640, Asn644, **Asn648**, Lys660, Gln663, **Asn666**, Lys670, Thr673, Gln674, Lys677, **Asn913**, Lys914, Asn937, Lys939, Asp940, Arg942	Arg81-Thr135^O^ (HB) Lys93-Asp612 (SB) Glu97^O^-Lys677 (HB) Glu100-Gln674 (HB) Lys205^O^-Arg629 (HB) Glu244-**Asn666** (HB) Lys317-**Asn648** (HB) Lys323-Asp940 (SB) Glu358-Lys939 (SB)

Glycosylated Asn residues of ACE are highlighted in bold. Asn residues that could potentially be glycosylated in native human ACE and not glycosylated in the recombinant ACE used for in silico experiments are highlighted in red. HB—hydrogen bond; SB—salt bridge; the superscripts O and HN denote amino acids in which the backbone atoms participate in the interaction.

**Table 2:** Structural characteristics of HSA–ACE complexes obtained by molecular dynamics simulation: the quantity of atoms in the HSA and ACE molecules forming close contacts between the proteins (at a distance of no more than 3.5 Å) at the end-point of the simulation, the main amino acids of HSA and ACE involved in these contacts and the amino acids involved in specific interactions (hydrogen bonds and salt bridges) and their lifetime as a percentage of the total simulation length (100 ns).

Complex # Quantity of Interacting Atoms, HSA/ACE	HSA	ACE	Specific Interactions HSA–ACE and Their Lifetime
4 278/259	Asp1, His9, Lys12, Asp13, Glu16, Glu17, Asn18, Lys20, Gln33, Pro35, Glu37, Asp38, Lys41, Asn44, Glu45, Glu48, Thr52, Val54, Ala55, Asp56, Glu57, Leu80, Thr83, Tyr84, Asn111, Leu112, Pro113, Asp129, Asn130, Glu131, Glu132, Leu135, Lys159, Lys162, Glu280, Pro282, Ala504, Glu505, Thr508, His510, Asp512, Glu565, Thr566, Ala569, Gly572, Lys573, Gln580	Pro141, Asn145, Ala148, Ser149, Arg235, Arg236, Thr280, Ser281, Leu284, Glu320, Lys321, Arg350, Thr352, Arg413, Lys622, Glu625, Arg629, **Asn666**, Lys670, Thr673, Gln674, Lys893, Thr900, Pro906, Pro908, Pro909, Glu910, Trp912, **Asn913**, Lys914, Lys939, Lys971, Lys1132	Asp1^NH^-Glu320 (SB, 29%) Asp13-Ser281 (HB, 75%) Glu17-Tyr265 (HB, 65%) Glu17-Asn423 (HB, 18%) Lys20-Asp266 (SB, 86%) Glu37-Arg236 (SB, 66%) Glu37-Lys622 (SB, 19%) Asp38-Lys622 (SB, 91%) Lys41-Asp616 (SB, 17%) Ala55^O^-Arg350 (HB, 67%) Glu57-Arg350 (SB, 76%) Thr79-Glu619 (HB, 5%) Asn111^O^-Arg629 (HB, 5%) His128^O^-Asn588 (HB, 30%) Asp129-Arg235 (SB, 96%) Lys159-Asp273 (SB, 63%) Lys162-Asp273 (SB, 89%) Glu280-Arg413 (SB, 67%) Lys286-Asp412 (SB, 11%) Glu505-Lys914 (SB, 71%) Glu505^O^-Lys939 (HB, 43%) Asp512-Lys971 (SB, 86%) Glu565-Lys1132 (SB, 34%)
7 198/202	Gln33, Pro35, Phe36, Glu37, Asp38, Lys41, Thr68, Asp72, Thr79, Leu80, Arg81, Glu82, Thr83, Tyr84, Glu86, Asp89, Ala92, Lys93, Gln94, Pro113, Arg114, Val116, Arg117, Glu119, Thr125, Asp129, Lys137, Arg144, Ala569	Arg240, Thr478, **Asn480**, Asp605, Arg676, Lys693, Lys689, Tyr789, Asp791, Asp794, Arg797, Ser798, Thr802, Pro803, Ser804, Glu806, Gln807, Glu810, Arg811, Gln814, Glu815, Gln817, Pro818, Arg1046, Asp1049, Ser1051, Lys1067, Thr1120, Pro1193, Tyr1195	Gln33^O^-Ser804 (HB, 80%) Cys34^O^-Gln807 (HB, 27%) Phe36^HN^-Glu806 (SB, 22%) Glu37-Arg1046 (SB, 83%) Asp38-Arg797 (SB, 98%) Lys41-Asp1049 (SB, 99%) Thr79-Asp794 (HB, 6%) Arg81-Asp605 (SB, 85%) Glu82-Lys689 (SB, 61%) Thr83-Ser798 (HB, 23%) Glu86-Arg240 (SB, 98%) Asp89-Arg240 (SB, 80%) Glu97-Thr478^HN^ (HB, 36%) Arg114-Glu815 (SB, 39%) Arg117^O^-Gln814 (HB, 46%) Asp129-Lys1067 (SB, 48%) Lys137-Glu810 (SB, 68%) Lys205-Glu596 (SB, 11%) Asp512-Arg1137 (SB, 7%) Glu565-Lys1132 (SB, 6%)
6 120/90	Asn130, Glu131, Glu132, Lys159, Arg160, Lys162, Thr166, Glu167, Gln170, Lys174, Lys181, Glu184, Leu185, Glu277, Glu280, Pro282, Lys432, Lys439	**Asn45**, Ile46, Thr47, **Asn117**, Arg120, Ile121, Thr124, Lys126, **Asn131**, Lys132, Thr133, Ala134, Thr135, Gly325, Glu327, Arg350	Lys159-Glu327 (SB, 74%) Lys162-Ile46^O^ (HB, 58%) Glu167-Lys126 (SB, 94%) Glu184-Lys132 (SB, 37%) Glu277-Thr144 (HB, 7%) Glu277-Arg350 (SB, 44%) Glu280-Arg350 (SB, 74%) Glu297-Arg236 (SB, 8%) Tyr334-Glu626 (HB, 14%) Tyr334-Arg629 (HB, 5%) Tyr341-Glu626 (HB, 16%)

Glycosylated Asn residues of ACE are highlighted in bold. Asn residues that could potentially be glycosylated in native human ACE and not glycosylated in the recombinant ACE used for in silico experiments are highlighted in red. HB—hydrogen bond; SB—salt bridge; the superscripts O and HN denote amino acids in which the backbone atoms participate in the interaction.

## Data Availability

The data presented in this study are available from the corresponding authors upon reasonable request.

## Supplementary Materials

The following supporting information can be downloaded at: https://www.mdpi.com/article/10.3390/ijms251910260/s1.

## Abbreviations

ACE	angiotensin I-converting enzyme;
AD	Alzheimer’s Disease;
AF3	AlphaFold 3;
FA	fatty acids;
FcRn	neonatal Fc receptor;
HB	hydrogen bond;
HSA	human serum albumin;
MD	molecular dynamics;
RAAS	renin–angiotensin–aldosterone system;
RMSD	root means square deviation;
SB	salt bridge;
VMD	Visual Molecular Dynamics
