# Supplementary material for: Computational Study of Molecular Mechanism for the Involvement of Human Serum Albumin in the Renin–Angiotensin–Aldosterone System
Source: Int J Mol Sci. 2024 Sep 24;25(19):10260. doi: 10.3390/ijms251910260 (PMC11476573; doi:10.3390/ijms251910260)
Supplement: Supplementary file 1 [file ijms-25-10260-s001.zip › ijms-3196233-supplementary.pdf]

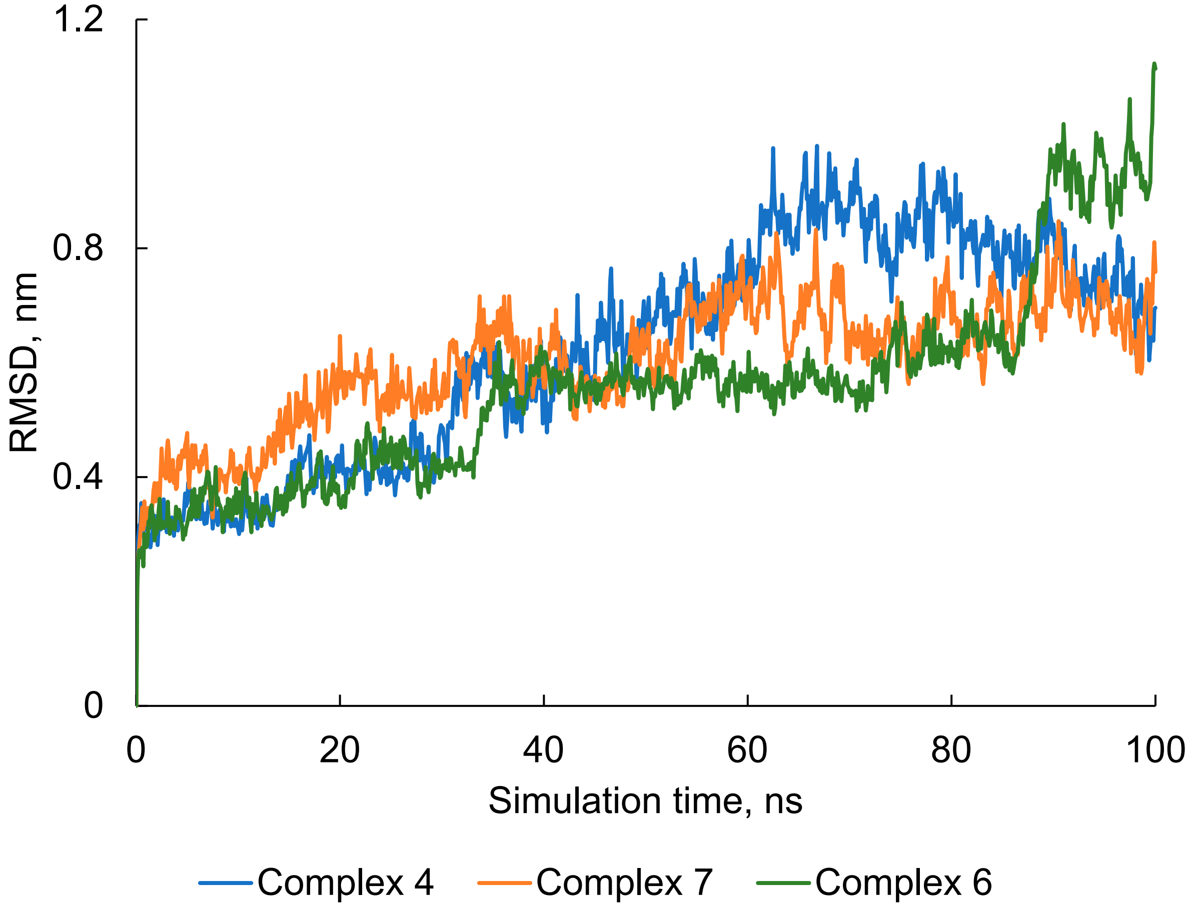

Figure S1. Root means square deviation (RMSD) of the Cα-atoms of the strongest complexes of human serum albumin (HSA) with angiotensin I-converting enzyme (ACE) according to molecular dynamics simulation.

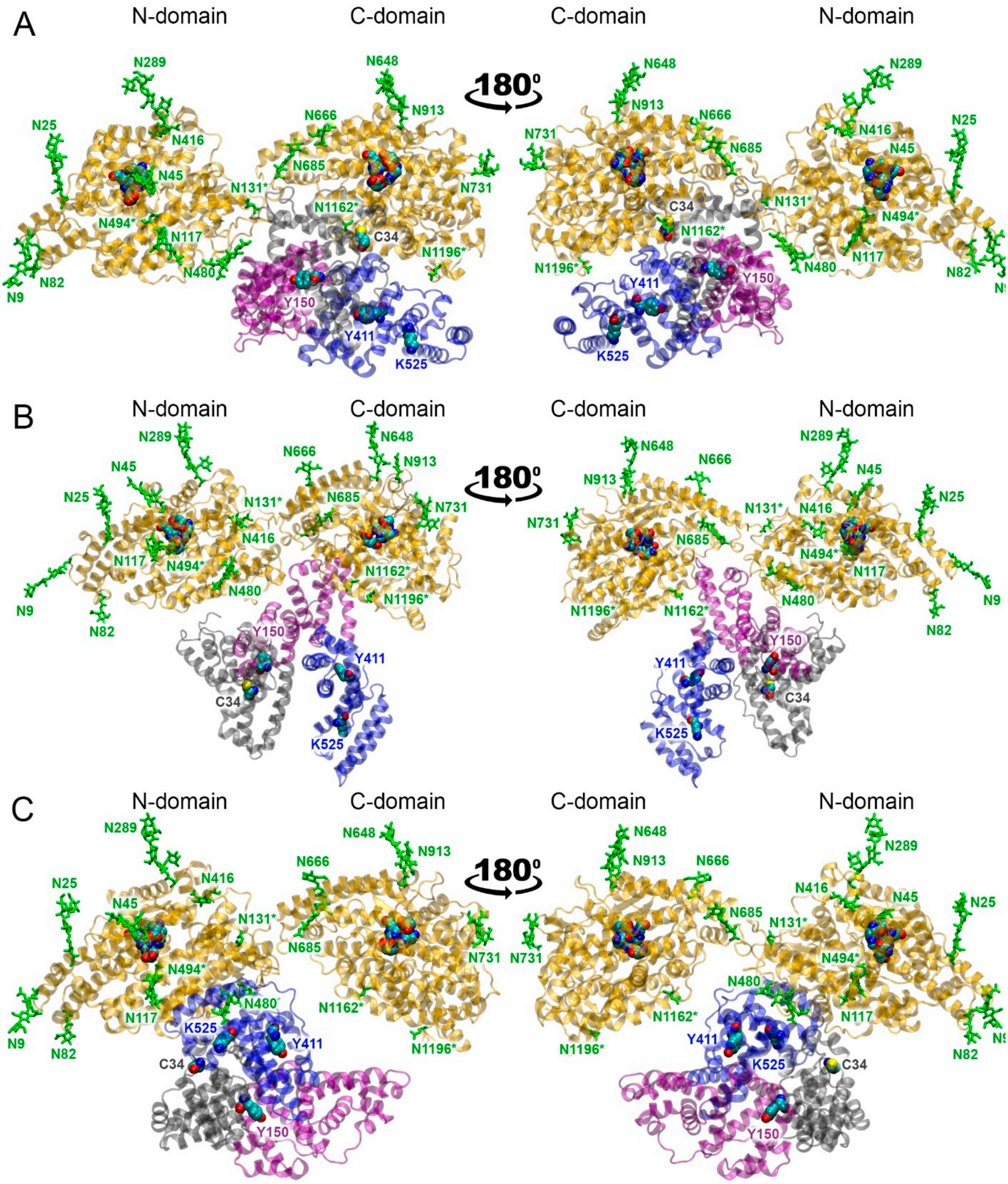

Figure S2. The most probable conformations of the HSA-ACE complex according to AlphaFold 3 (AF3). A – the leading complex with the maximum number of contacts between HSA and ACE (complex 1-AF). B – the second complex in the number of contacts between HSA and ACE (complex 2-AF). C – the third complex in the number of contacts between HSA and ACE (complex 3-AF). HSA domains DI, DII, and DIII are represented by gray, purple, and blue ribbon, respectively. The ACE molecule is represented by a yellow ribbon, the glycosylated ACE residues are shown with green sticks. The asterisk (*) symbols indicate asparagine residues that could potentially be glycosylated in native human ACE and were not glycosylated in the recombinant ACE used for *in silico* experiments. N- and C-domains of ACE are designated. The key amino acids of HSA and the active sites of ACE are shown as spheres (cyan, blue, red and yellow spheres represent carbon, nitrogen, oxygen and sulfur atoms, respectively). Hydrogen atoms are omitted for clarity.

Table S1. Structural characteristics of the three tightest HSA-ACE complexes obtained by AF3: quantity of atoms in HSA and ACE molecules forming close contacts between the proteins (at a distance of no more than 3.5 Å), the main amino acids of HSA and ACE involved in these contacts, as well as the amino acids involved in specific interactions (hydrogen bonds and salt bridges).

**Complex #**<br><br>**Quantity of interacting atoms, HSA / ACE**	**HSA**	**ACE**	**Specific interactions HSA-ACE**
1-AF<br><br>81 / 95	Glu86, Asp89, Lys93, Glu97, Gln104, Glu321, Thr467, Pro468, Asp471, Val498, Lys500, Glu501	Thr478, Gln706, Glu707, Lys775, Glu778, Leu779, Gln782, Arg785, Arg1046, Ser1051, Lys1054, Asn1056, Lys1067, Pro1193, Tyr1195, Asn1196	Lys93-Glu707 (SB)<br>Lys93-Glu778 (SB)<br>Lys93-Gln782 (HB)<br>Asp471-Lys1067 (SB)
2-AF<br><br>73 / 86	Asp1, Ala2, Lys4, Ala229, Thr236, Lys240, Glu321, Ala322, Val325, Met329	Arg231, His234, Arg235, Leu244, Arg245, Pro455, Arg458, Asp462, Tyr465, Pro595, Glu596	Glu321-Arg231 (SB)<br>Glu321^{O}-His234 (HB)<br>Glu321-Arg235 (SB)
3-AF<br><br>68 / 74	Arg81, Glu82, Tyr84, Gly85, Glu86, Asp89, His105, Asn111, Lys500, Glu501	Lys132, Arg453, Ser457, Arg458, Phe461, Tyr465, **Asn480**, Glu481, Tyr597, Gln598	Arg81-Tyr465 (HB)<br>Glu82-Arg453 (SB)<br>Glu86-Gln598 (HB)<br>Glu501-Lys132 (SB)

Glycosylated Asn residues in of ACE are highlighted bold; Asn residues that could potentially be glycosylated in native human ACE and not glycosylated in the recombinant ACE used for *in silico* experiments are highlighted red; HB – hydrogen bond; SB – salt bridge; the superscripts O denotes amino acids in which the backbone atoms participate in the interaction.
